# Identifying Conditions With High Prevalence, Cost, and Variation in Cost in US Children’s Hospitals

**DOI:** 10.1001/jamanetworkopen.2021.17816

**Published:** 2021-07-26

**Authors:** Peter J. Gill, Mohammed Rashidul Anwar, Thaksha Thavam, Matt Hall, Jonathan Rodean, Sunitha V. Kaiser, Rajendu Srivastava, Ron Keren, Sanjay Mahant

**Affiliations:** 1 Department of Pediatrics, Institute for Health Policy, Management and Evaluation, University of Toronto, Toronto, Canada; 2 Child Health Evaluative Sciences, Research Institute, The Hospital for Sick Children, Toronto, Canada; 3 Children’s Hospital Association, Lenexa, Kansas; 4 Department of Pediatrics and Department of Epidemiology and Biostatistics, University of California, San Francisco; 5 Philip R. Lee Institute for Health Policy Studies, San Francisco, California; 6 Department of Pediatrics, University of Utah, Primary Children’s Hospital, Salt Lake City; 7 Healthcare Delivery Institute, Intermountain Healthcare, Salt Lake City, Utah; 8 Department of Pediatrics, The Children's Hospital of Philadelphia, Philadelphia, Pennsylvania

## Abstract

**Importance:**

Identifying high priority pediatric conditions is important for setting a research agenda in hospital pediatrics that will benefit families, clinicians, and the health care system. However, the last such prioritization study was conducted more than a decade ago and used *International Classification of Diseases, Ninth Revision, Clinical Modification* (*ICD-9-CM*) codes.

**Objectives:**

To identify conditions that should be prioritized for comparative effectiveness research based on prevalence, cost, and variation in cost of hospitalizations using contemporary data at US children’s hospitals.

**Design, Setting, and Participants:**

This retrospective cohort study of children with hospital encounters used data from the Pediatric Health Information System database. Children younger than 18 years with inpatient hospital encounters at 45 tertiary care US children’s hospitals between January 1, 2016, and December 31, 2019, were included. Data were analyzed from March 2020 to April 2021.

**Main Outcomes and Measures:**

The condition-specific prevalence and total standardized cost, the corresponding prevalence and cost ranks, and the variation in standardized cost per encounter across hospitals were analyzed. The variation in cost was assessed using the number of outlier hospitals and intraclass correlation coefficient.

**Results:**

There were 2 882 490 inpatient hospital encounters (median [interquartile range] age, 4 [1-12] years; 1 554 024 [53.9%] boys) included. Among the 50 most prevalent and 50 most costly conditions (total, 74 conditions), 49 (66.2%) were medical, 15 (20.3%) were surgical, and 10 (13.5%) were medical/surgical. The top 10 conditions by cost accounted for $12.4 billion of $33.4 billion total costs (37.4%) and 592 815 encounters (33.8% of all encounters). Of 74 conditions, 4 conditions had an intraclass correlation coefficient (ICC) of 0.30 or higher (ie, major depressive disorder: ICC, 0.49; type 1 diabetes with complications: ICC, 0.36; diabetic ketoacidosis: ICC, 0.33; acute appendicitis without peritonitis: ICC, 0.30), and 9 conditions had an ICC higher than 0.20 (scoliosis: ICC, 0.27; hypertrophy of tonsils and adenoids: ICC, 0.26; supracondylar fracture of humerus: ICC, 0.25; cleft lip and palate: ICC, 0.24; acute appendicitis with peritonitis: ICC, 0.21). Examples of conditions high in prevalence, cost, and variation in cost included major depressive disorder (cost rank, 19; prevalence rank, 10; ICC, 0.49), scoliosis (cost rank, 6; prevalence rank, 38; ICC, 0.27), acute appendicitis with peritonitis (cost rank, 13; prevalence rank, 11; ICC, 0.21), asthma (cost rank, 10; prevalence rank, 2; ICC, 0.17), and dehydration (cost rank, 24; prevalence rank, 8; ICC, 0.18).

**Conclusions and Relevance:**

This cohort study found that major depressive disorder, scoliosis, acute appendicitis with peritonitis, asthma, and dehydration were high in prevalence, costs, and variation in cost. These results could help identify where future comparative effectiveness research in hospital pediatrics should be targeted to improve the care and outcomes of hospitalized children.

## Key Points

**Question:**

What conditions have the highest prevalence, cost, and variation in cost in hospital pediatrics?

**Findings:**

This cohort study included 2 882 490 inpatient hospital encounters of children from US children’s hospitals to identify conditions with high prevalence, cost, and interhospital variation in cost. Examples of conditions that were identified as having high prevalence, cost, and variation in cost included major depressive disorder, scoliosis, acute appendicitis with peritonitis, asthma, and dehydration.

**Meaning:**

The findings from this cohort study could inform funders and researchers of areas at which research in hospital pediatrics should be targeted to improve the evidence base and outcomes of hospitalized children.

## Teaser

This cohort study examines prevalence, cost, and variation in cost of hospitalizations in US pediatric hospitals.

## Introduction

The hospital is a high-cost, resource-intensive setting where there is increasing pressure to provide safe and high-quality care efficiently for children.^1,2^ Despite the high cost of hospital care, there are still many areas in pediatric hospital care that lack high-quality evidence, including the treatment of children with common conditions and those with complex health care needs.^3,4^ Comparative effectiveness research, which aims to determine which clinical and health care delivery strategies are most effective in real-word settings, is important to inform practice, reduce unnecessary practice variation, and improve health outcomes.^5^

Prioritizing topics for comparative effectiveness research in hospital pediatrics is an important step to develop a research agenda that will benefit children and families, clinicians, and the health care system. A 2012 analysis by Keren et al^6^ identified high-priority pediatric conditions for comparative effectiveness research using data on prevalence, cost, and variation in cost of hospitalizations in US children’s hospitals. However, the study by Keren et al^6^ included data from 2004 to 2009, which are now more than a decade old. The study also used the *International Classification of Diseases, Ninth Revision, Clinical Modification* (*ICD-9-CM*)^7^ codes to identify the primary discharge diagnosis, but in 2015, the US transitioned to *International Statistical Classification of Diseases, Tenth Revision, Clinical Modification* (*ICD-10-CM*),^8,9^ which has improved specificity and increased granularity.^8,10^ The Institute of Medicine recommends setting the prioritization criteria every 5 years and having the priority-setting cycle (ie, producing a rank-order list of conditions to be prioritized) every 3 years.^11^ A 2011 review by Dubois and Graff,^12^ which developed a framework for setting priorities for research, also suggested updating research prioritization using the same frequency. Over time, improvements in health care delivery, technologies, and procedures may affect costs, variation in care, and treatment choices.^12^ Therefore, it is important to update the prioritization regularly.^12^

In this study, we updated the research prioritization agenda in hospital pediatrics using a similar approach to Keren et al,^6^ using the *ICD-10-CM* system applied to contemporary data. We aimed to identify conditions that should be prioritized for comparative effectiveness research in hospital pediatrics. The specific objectives were to describe the condition-specific prevalence, cost, and variation in cost of pediatric hospitalizations and rank order conditions according to prevalence and cumulative cost, and identify conditions with high prevalence, cost, and variation in cost as targets for prioritization for research in hospitalized children.

## Methods

This cohort study was approved by the research ethics board of the Hospital for Sick Children, and the requirement for informed consent was waived because patient-level data were deidentified. This study followed the Strengthening the Reporting of Observational Studies in Epidemiology (STROBE) reporting guideline.

### Design and Data Source

We conducted a retrospective cohort study using data from the Pediatric Health Information System (PHIS), an administrative database containing hospitalization data from 50 tertiary care children’s hospitals developed by the Children’s Hospital Association, located in Lenexa, Kansas. The PHIS database includes detailed data on demographics, diagnosis codes, service locations, procedures, and charges. The hospital billing data are mapped to a common set of clinical transaction codes, which are further categorized into imaging studies, clinical services, laboratory tests, pharmacy, supplies, and room charges. Data are subjected to several checks of reliability and validity and processed into data quality reports.

### Study Population

The study population included children younger than 18 years with an inpatient hospital encounter (ie, inpatient and observation encounters in the PHIS database) between January 1, 2016, and December 31, 2019. We excluded hospitals that had incomplete billing data for the study period. We also excluded encounters for children with an *ICD-10-CM* primary discharge diagnosis code for normal newborn births, with external cause codes, with invalid diagnosis codes, with missing billing or cost data, and those from ambulatory surgery. We also excluded extreme cost outliers (defined as the top 1% of standardized cost within each condition) to minimize potential data errors and unusual clinical encounters, similar to the study by Keren et al.^6^

### Patient, Encounter, and Hospital Characteristics

Patient characteristics included age (<30 days, ≥30 days to <1 year, 1-4 years, 5-12 years, and 13-17 years), sex, race/ethnicity (categorized as non-Hispanic White, non-Hispanic Black, Hispanic, and other [including American Indian, Alaska Native, Asian, multiracial, Native Hawaiian, Pacific Islander, missing data, and other]), and primary payer (ie, government, private, or other). Race/ethnicity was self-identified by parents and families using each hospital’s classification system and was included as a characteristic to describe children with encounters. Median zip code household income as a percentage of the federal poverty level^13^ was determined for each encounter to understand the socioeconomic distribution of the cohort. We used Rural-Urban Commuting Area codes to determine the rural-urban classification of each patient’s residence into metropolitan, micropolitan, small town, and rural areas.^14,15,16^ We determined the number of complex chronic conditions (CCCs)^17^ present (0, 1, 2, or ≥3)^18^ based on a 1-year lookback or until birth if younger than 1 year from each hospital encounter date. We also identified the patient type based on the encounter location (ie, inpatient or observation unit) and determined the length of stay (in days). For the hospital characteristics, we identified the census region (Midwest, Northwest, South, or West), and the median volume of inpatient encounters per year.

### Pediatric Clinical Classification System

We classified the primary discharge diagnosis code for all encounters using the Pediatric Clinical Classification System (PECCS).^19^ The PECCS (developed using the Healthcare Cost and Utilization Project Clinical Classifications Software^20^ and the pediatric diagnosis code grouper used by Keren et al^6^) classifies all 72 446 *ICD-10-CM* diagnosis codes into 834 clinically meaningful categories to help identify specific pediatric conditions, including treatments (eg, chemotherapy). Conditions were further divided into medical, surgical, or medical/surgical based on the percentage of encounters with a surgical *ICD-10-CM* Procedure Coding System procedure or a *Current Procedural Terminology* code. Conditions with less than 30% of encounters with a surgical procedure code were classified as medical, more than 70% as surgical, and between 30% and 70% as medical/surgical.

### Calculation of Standardized Cost

Since cost of individual items (eg, laboratory tests, imaging, room charges) varied between hospitals, we used standard costs of those items across hospitals. The Cost Master Index, calculated yearly and maintained by the Children’s Hospital Association, provides the standard unit costs for all individual items. For each item billed in a given year, the item’s cost is determined using the item’s charge, the hospital- and department-specific ratio of cost to charges, and the number of billed units for the item. Then, the within-hospital median of costs for the specific item is calculated. Finally, the across-hospital median of the within-hospital median cost for the item provides the standardized unit cost for the specific item during a specific year.^6,21^

Hospitalization costs were used as a surrogate measure of the volume of resources used for the encounters.^6^ These costs were standardized to eliminate the high interhospital variation in item costs.^6^ For each condition, we calculated the cost of an encounter by multiplying the number of units for each clinical transaction code item by the item’s standardized cost. We then summed the standardized costs of each line item for that encounter. We defined each clinical transaction code item’s standardized cost by the Cost Master Index,^6^ and adjusted costs for inflation to 2019 US dollars using the Consumer Price Index for hospital services.^22^ When we use the term *cost*, we are referring to the calculated standardized cost.

### Outcome Measures

We determined the condition-specific prevalence rank for each hospital condition based on the number of encounters over the study period. For each condition, we determined the condition-specific cost rank based on the cumulative cost of hospital encounters over the study period. The condition-specific variation in cost per encounter across hospitals was also determined over the study period.

### Statistical Analysis

We determined the mean cost per inpatient hospital encounter for each hospital condition. We then determined the variation in cost of hospitalization by condition for the 50 most prevalent and 50 most costly conditions, focusing on their cost per encounter, across hospitals. The condition-specific variation in cost across hospitals was adjusted for known drivers of variation in cost to minimize confounding from other factors that may bias the magnitude of variation in cost per encounter across hospitals.^6,23,24,25^ These included age, sex, race/ethnicity, patient type, and number of CCCs present (0, 1, 2, or ≥3). Rural-Urban Commuting Area, primary payer, and income were not included owing to high multicollinearity. The variation in cost per encounter was assessed using 2 methods presented in the study by Keren et al.^6^ First, for number of outlier hospitals, we counted the number of hospitals with more than 30% of their encounters for each condition in either the highest or lowest quintile of cost per encounter. Second, for intraclass correlation coefficient (ICC), the amount of variation in costs (cost per encounter) for each condition across hospitals was divided by the total variation in the cost per encounter (ie, sum of the within- and across hospital variation of costs). ICC was calculated using a mixed-effects model, with hospital as a random intercept, and patient characteristics as fixed effects.^6^

Additional analyses were performed to determine the 25 most prevalent and 25 most costly conditions for children with CCCs^17^ vs children without. These analyses were conducted because children with medical complexity have a low prevalence but high total health care costs^26^ and have unique disease management and health care needs. Analyses were conducted using SAS statistical software version 9.4 (SAS Institute). Data were analyzed from March 2020 to April 2021.

## Results

There were 5 555 810 hospital encounters in children’s hospitals between January 1, 2016, to December 31, 2019. After applying the exclusion criteria, 2 882 490 inpatient hospital encounters across 45 children’s hospitals were included (eFigure in Supplement 1).

### Patient, Encounter, and Hospital Characteristics

Of the 2 882 490 inpatient encounters, 2 188 278 (75.9%) were children aged 1 year or older, the median (interquartile range [IQR]) age was 4 (1-12) years, and 1 554 024 (53.9%) were boys (Table 1). Children with 1 or more CCC accounted for 1 132 532 encounters (39.3%). Over half of the encounters (1 551 117 encounters [53.8%]) were of children with a median household income less than 200% of the US federal poverty level, and 1 623 655 encounters (56.3%) were in children covered by government insurance. A total of 1 852 308 encounters (64.3%) were owing to medical conditions, 578 230 encounters (20.1%) were owing to surgical conditions, and 451 952 encounters (15.7%) were owing to medical/surgical conditions. The median (IQR) length of stay was 3 (2-5) days, and the median hospital volume of inpatient encounters per year was 15 067 (9510-19 514) encounters.

**Table 1.** Patient, Encounter, and Hospital Characteristics for Children With Inpatient Hospital Encounters at 45 US Children’s Hospitals, 2016 to 2019

Characteristic	No. (%)
**Patient characteristics**	
No. of encounters	2 882 490
Age	
Median (IQR), y	4 (1-12)
<30 d	235 311 (8.2)
≥30 d to <1 y	458 901 (15.9)
1-4 y	747 767 (25.9)
5-12 y	825 463 (28.6)
13-17 y	615 048 (21.3)
Sex	
Boys	1 554 024 (53.9)
Girls	1 327 836 (46.1)
Missing	630 (<0.1)
RUCA designation	
Metropolitan	2 403 433 (83.4)
Micropolitan	218 918 (7.6)
Small town	117 665 (4.1)
Rural	68 136 (2.4)
Missing	74 338 (2.6)
Complex chronic conditions present, No.	
0	1 749 958 (60.7)
1	687 031 (23.8)
2	276 918 (9.6)
≥3	168 583 (5.8)
Median household income for zip code, % of federal poverty level^a^	
<150	680 962 (23.6)
150-199	870 155 (30.2)
200-249	581 584 (20.2)
≥250	675 507 (23.4)
Missing	74 282 (2.6)
Primary payer	
Government	1 623 655 (56.3)
Private	1 114 087 (38.7)
Other	98 594 (3.4)
Missing	46 154 (1.6)
Race/ethnicity	
Non-Hispanic White	1 385 457 (48.1)
Non-Hispanic Black	525 281 (18.2)
Hispanic	595 067 (20.6)
Other^b^	376 685 (13.1)
Hospital encounter characteristics	
Condition type	
Medical	1 852 308 (64.3)
Medical/surgical	451 952 (15.7)
Surgical	578 230 (20.1)
Patient type	
Inpatient	1 982 571 (68.8)
Observation	899 919 (31.2)
Length of stay, median (IQR), d	3 (2-5)
**Hospital characteristics**	
No. of hospitals	45
Region	
Midwest	12 (26.7)
Northwest	5 (11.1)
South	17 (37.8)
West	11 (24.4)
Volume of inpatient encounters per year, median (IQR), No.^c^	15 067 (9510-19 514)

Abbreviations: IQR, interquartile range; RUCA, Rural-Urban Commuting Area.

^a^ Median income is based on the United States Federal Poverty Level guidelines.

^b^ Other race/ethnicity includes American Indian, Alaska Native, Asian, multiracial, Native Hawaiian, Pacific Islander, missing, and other.

^c^ Includes inpatient or observation unit encounters.

### Prevalence and Cost

Table 2 shows the 50 most prevalent and 50 most costly hospital conditions, with a total of 74 different conditions, sorted by total cost over the 4-year period. Of 74 conditions, 49 (66.2%) were medical, 15 (20.3%) were surgical, and 10 (13.5%) were medical/surgical. The top 10 conditions by cost accounted for $12.4 billion of $33.0 billion total costs (37.4%) and 592 815 encounters (33.8% of all encounters). Extreme immaturity conditions (ie, birth weight 500-749 g) had the highest cost per encounter, at $382 910 (95% CI, $368 084-$397 736). There were also 2 mental health conditions observed in the top 50 most prevalent and 50 most costly hospital conditions: major depressive disorder (cost rank, 19; prevalence rank, 10; ICC, 0.49) and suicide and intentional self-inflicted injury (cost rank, 57; prevalence rank, 20; ICC, 0.19).

**Table 2.** Prevalence, Cost, and Variation in Cost for the 50 Most Prevalent and 50 Most Costly Inpatient Hospital Conditions at 45 US Children’s Hospitals From 2016 to 2019^a^

Condition	Type	Rank based on		Total encounters, No.	Standardized cost, $		ICC^b^	Outlier hospitals, No.^b^	
		Cost	Prevalence		Per encounter, mean (95% CI)	Total, millions		Low	High
Respiratory failure	Medical	1	4	79 496	29 861 (29 511-30 212)	2374	0.07	7	9
Chemotherapy	Medical	2	5	70 804	24 543 (24 318-24 768)	1738	0.14	8	6
Septicemia	Medical	3	16	31 318	48 931 (48 042-49 820)	1532	0.06	2	8
Bronchiolitis	Medical	4	1	143 379	8609 (8552-8667)	1234	0.09	11	4
Pneumonia	Medical	5	3	83 884	13 694 (13 559-13 830)	1149	0.10	8	6
Scoliosis	Surgical	6	38	16 829	62 395 (61 880-62 911)	1050	0.27	10	11
Respiratory distress syndrome in newborn	Medical	7	70	8464	112 484 (109 065-115 903)	952	0.19	8	9
Hypoplastic left heart syndrome	Medical/surgical	8	109	5373	155 749 (149 604-161 894)	837	0.10	11	6
Complications of surgical procedures or medical care	Medical/surgical	9	13	35 594	21 137 (20 807-21 467)	752	0.07	6	7
Asthma	Medical	10	2	117 674	6293 (6261-6324)	740	0.17	12	7
Respiratory failure of newborn	Medical	11	80	6855	105 320 (100 122-110 518)	722	0.12	6	10
Extreme immaturity (birth weight, 500-749 g)	Medical	12	277	1745	382 910 (368 084-397 736)	668	0.06	16	6
Acute appendicitis with peritonitis	Surgical	13	11	39 866	16 043 (15 927-16 158)	640	0.21	7	10
Transposition of great vessels	Medical/surgical	14	117	5026	124 821 (120 896-128 746)	627	0.10	10	7
Tetralogy of fallot	Medical/surgical	15	85	6558	91 978 (88 890-95 066)	603	0.06	8	6
Extreme immaturity (birth weight, 750-999 g)	Medical	16	252	1934	305 911 (297 748-314 074)	592	0.05	14	8
Seizures with and without intractable epilepsy	Medical	17	6	57 820	9944 (9837-10 052)	575	0.11	12	5
Congestive heart failure (nonhypertensive)	Medical	18	182	3158	179 930 (168 974-190 887)	568	0.10	6	7
Major depressive disorder	Medical	19	10	46 058	10 347 (10 287-10 406)	477	0.49	21	12
Sepsis of newborn	Medical	20	104	5749	81 611 (78 020-85 203)	469	0.07	9	5
Specified conditions originating in perinatal period	Medical	21	29	22 172	20 094 (19 429-20 759)	446	0.10	7	5
Acute lymphoid leukemia without remission	Medical	22	92	6197	71 577 (69 768-73 386)	444	0.13	10	6
Coarctation of aorta or interrupted aortic arch	Surgical	23	113	5227	82 204 (79 297-85 111)	430	0.08	9	6
Dehydration	Medical	24	8	54 873	7639 (7565-7713)	419	0.18	13	8
Extreme immaturity (birth weight, 1000-1249 g)	Medical	25	244	1991	209 896 (204 669-215 124)	418	0.01	11	12
Bronchopulmonary dysplasia	Medical	26	306	1496	278 224 (257 006-299 443)	416	0.11	16	7
Anomalies of diaphragm, congenital	Surgical	27	254	1931	212 366 (198 449-226 284)	410	0.09	13	4
Cellulitis	Medical	28	9	54 577	7253 (7202-7304)	396	0.12	11	9
Necrotizing enterocolitis	Medical/surgical	29	285	1659	237 476 (224 315-250 637)	394	0.03	7	5
Partial epilepsy with and without intractable epilepsy	Medical	30	19	28 920	13 485 (13 266-13 705)	390	0.13	8	7
Endocardial cushion defects	Surgical	31	158	3841	100 320 (96 034-104 606)	385	0.09	11	9
Intracranial injury	Medical	32	44	13 561	28 412 (27 617-29 206)	385	0.07	8	6
Cystic fibrosis	Medical	33	64	9530	40 408 (39 737-41 079)	385	0.15	14	8
Neutropenia	Medical	34	33	19 580	19 583 (19 237-19 929)	383	0.11	9	6
Complication of device, implant, or graft	Surgical	35	40	16 209	23 593 (23 163-24 022)	382	0.07	5	2
Acute appendicitis without peritonitis	Surgical	36	12	38 787	9434 (9385-9482)	366	0.30	12	14
Gastroschisis and exomphalos	Surgical	37	239	2114	172 017 (162 628-181 407)	364	0.07	6	9
Ventricular septal defect	Medical/surgical	38	93	6192	57 431 (55 992-58 870)	356	0.11	10	9
Other congenital anomalies	Surgical	39	49	12 054	28 631 (27 933-29 328)	345	0.04	9	4
Preterm newborn	Medical	40	101	5859	58 644 (56 821-60 467)	344	0.11	9	19
Pericarditis, endocarditis, myocarditis, and cardiomyopathy	Medical	41	144	4306	78 706 (73 897-83 516)	339	0.06	5	5
Other nervous system disorders	Medical	42	32	20 054	16 017 (15 708-16 327)	321	0.07	9	3
Preterm infant (birth weight, 1250-1499 g)	Medical	43	234	2165	143 155 (139 715-146 594)	310	0.03	9	11
Sickle cell disease with crisis	Medical	44	25	23 261	13 298 (13 145-13 451)	309	0.19	9	11
Fracture of lower limb	Surgical	45	23	25 191	12 197 (12 069-12 326)	307	0.10	5	9
Preterm infants (birth weight, 2000-2499 g)	Medical	46	84	6646	46 134 (45 305-46 964)	307	0.06	7	16
Urinary tract infections	Medical	47	14	33 918	8998 (8908-9089)	305	0.10	11	8
Diabetic ketoacidosis	Medical	48	17	30 619	9516 (9430-9602)	291	0.33	10	10
Gastroenteritis, infectious	Medical	49	15	32 531	8777 (8675-8879)	286	0.13	13	9
Intrauterine hypoxia and birth asphyxia	Medical	50	155	3870	73 398 (71 165-75 630)	284	0.10	9	9
Hypertrophy of tonsils and adenoids	Surgical	52	7	54 914	5017 (4996-5039)	276	0.26	7	19
Feeding difficulties and mismanagement	Medical/surgical	54	46	13 323	18 108 (17 652-18 563)	241	0.09	10	7
Suicide and intentional self-inflicted injury	Medical	57	20	28 905	8138 (8040-8237)	235	0.19	6	8
Viral infection	Medical	63	21	28 007	8130 (8027-8233)	228	0.11	11	8
Constipation	Medical	73	22	25 717	7640 (7554-7726)	196	0.13	7	7
Failure to thrive	Medical	79	43	13 671	13 631 (13 348-13 914)	186	0.05	9	6
Skull and face fractures	Medical/surgical	81	36	18 218	10 043 (9852-10 234)	183	0.11	11	9
Headache; including migraine	Medical	84	31	20 115	8863 (8729-8996)	178	0.15	13	9
Sleep apnea	Surgical	87	35	18 933	9018 (8884-9151)	171	0.11	10	11
Other convulsions	Medical	90	18	29 275	5620 (5552-5688)	165	0.15	4	7
Cleft lip and palate	Surgical	91	41	15 271	10 690 (10 575-10 804)	163	0.24	8	13
Acute upper respiratory infection	Medical	93	27	22 995	6842 (6745-6940)	157	0.09	9	7
Supracondylar fracture of humerus	Surgical	97	28	22 371	6676 (6629-6724)	149	0.25	11	14
Fracture of upper limb	Surgical	99	37	17 251	8506 (8428-8583)	147	0.11	9	9
Fever of unknown origin	Medical	101	34	19 481	7212 (7114-7311)	140	0.13	11	6
Other lower respiratory disease	Medical	106	45	13 467	9970 (9688-10 252)	134	0.11	12	5
Influenza	Medical	109	39	16 595	7841 (7699-7983)	130	0.13	11	9
Gastroesophageal reflux and esophagitis	Medical/surgical	110	47	13 286	9747 (9580-9913)	129	0.09	10	6
Abdominal pain	Medical/surgical	111	30	20 268	6249 (6183-6314)	127	0.17	11	9
Epilepsy; convulsions	Medical	120	50	11 995	9774 (9533-10 014)	117	0.14	10	7
Neonatal hyperbilirubinemia	Medical	135	24	23 461	4450 (4394-4505)	104	0.15	12	8
Croup	Medical	152	26	23 157	3740 (3692-3787)	87	0.12	11	5
Type 1 diabetes with complications	Medical	159	48	12 174	6870 (6788-6953)	84	0.36	12	9
Allergic reactions	Medical	196	42	13 683	4709 (4628-4790)	64	0.13	13	11

Abbreviation: ICC, Intraclass correlation coefficient.

^a^ Includes inpatient or observation unit encounters.

^b^ ICC and number of outlier hospitals were calculated using standardized costs that were adjusted for age, sex, race/ethnicity, patient type, and number of complex chronic conditions present.

From the 74 most prevalent and/or costly conditions, major depressive disorder (ICC, 0.49), type 1 diabetes with complications (ICC, 0.36), diabetic ketoacidosis (ICC, 0.33), and acute appendicitis without peritonitis (ICC, 0.30) were 4 conditions with the highest degree of interhospital variability in cost per encounter using ICC. In total, there were 9 conditions that had an ICC higher than 0.20 (the additional 5 conditions were scoliosis: ICC, 0.27; hypertrophy of tonsils and adenoids: ICC, 0.26; supracondylar fracture of humerus: ICC, 0.25; cleft lip and palate: ICC, 0.24; and acute appendicitis with peritonitis: ICC, 0.21). When evaluating interhospital variation in cost using the outlier hospital analysis, more than half of the hospitals had a high proportion of high- or low-cost hospitalizations for 9 conditions (Table 2). Major depressive disorder had the highest number of outlier hospitals (33 cost outlier hospitals).

Conditions that were high in prevalence, cost, and variation in cost included, for example, major depressive disorder (cost rank, 19; prevalence rank, 10; ICC, 0.49), scoliosis (cost rank, 6; prevalence rank, 38; ICC, 0.27), acute appendicitis with peritonitis (cost rank, 13; prevalence rank, 11; ICC, 0.21), asthma (cost rank, 10; prevalence rank, 2; ICC, 0.17), and dehydration (cost rank, 24; prevalence rank, 8; ICC, 0.18). The Figure illustrates the top 25 costly conditions. Major depressive disorder (Figure, A) was highly prevalent, costly, and had the highest interhospital variability in cost per encounter of all medical conditions. Figure, B, represents 3 surgical and 4 medical/surgical conditions. Scoliosis and acute appendicitis with peritonitis were surgical conditions that were highly prevalent, costly, and with high interhospital variability.

**Figure.**
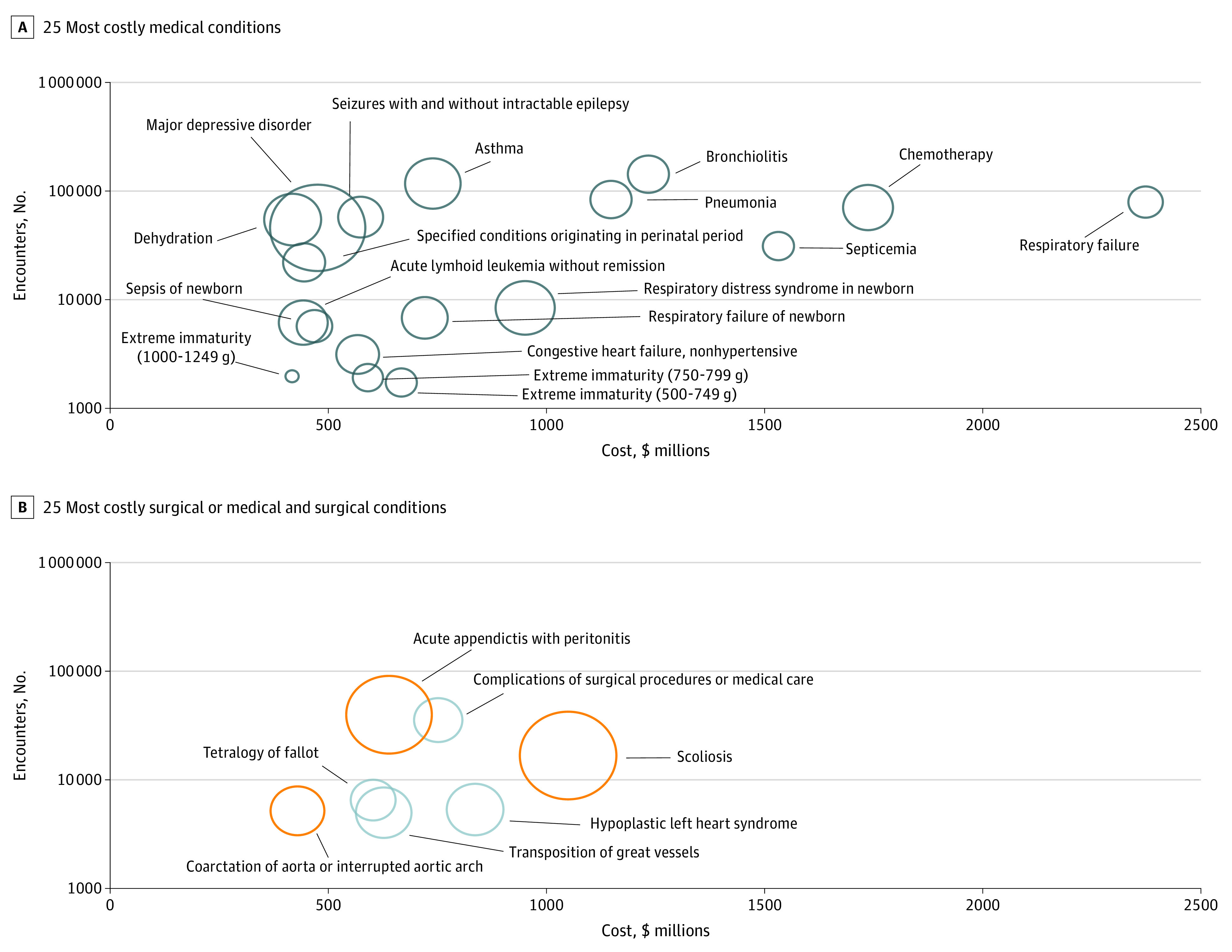
Prevalence, Cost, and Variation in Cost for the 25 Most Costly Conditions Data are derived from Pediatric Health Information System database spanning from January 1, 2016, to December 31, 2019. Bubble size indicates the interhospital variation in cost per encounter per condition (ie, larger bubble size means greater variation). B, orange bubbles indicate surgical conditions; grey bubbles indicate medical and surgical conditions

### Prevalence and Cost by Presence of Pediatric Complex Chronic Condition

Table 3 presents the volume of the 10 most prevalent conditions and cost of the 10 most costly (based on cumulative cost) conditions in children with a CCC vs those without. The 25 most prevalent and most costly conditions are reported in eTable 1 and eTable 2 in Supplement 1. The rank-order of the conditions differed between the 2 groups. In children with a CCC, the most prevalent and most costly conditions were chemotherapy and respiratory failure. However, in children without a CCC, bronchiolitis was the most prevalent and most costly condition. In children with a CCC, the 25 most costly conditions cost $15.8 billion, while in children without CCC they cost $8.3 billion. Furthermore, the cost per encounter for some of the top 25 costly conditions (eg, respiratory failure, pneumonia) that were present in both groups were 2- to 3-fold greater in children with a CCC.

**Table 3.** Comparison of 10 Most Prevalent and Costly Conditions in Children With and Without a Complex Chronic Condition at 45 US Children’s Hospitals, 2016 to 2019

Rank	Most prevalent conditions				Most costly conditions			
	Non-CCC	Total encounters, No.	CCC	Total encounters, No.	Non-CCC	Total standardized cost, $ in millions	CCC	Total standardized cost, $ in millions
1	Bronchiolitis	119 686	Chemotherapy	70 727	Bronchiolitis	877	Chemotherapy	1737
2	Asthma	108 642	Respiratory failure	33 981	Respiratory failure	707	Respiratory failure	1667
3	Pneumonia	55 468	Seizures with and without intractable epilepsy	31 313	Asthma	661	Septicemia	1217
4	Cellulitis	48 133	Pneumonia	28 416	Acute appendicitis with peritonitis	601	Hypoplastic left heart syndrome	837
5	Hypertrophy of tonsils and adenoids	46 813	Bronchiolitis	23 693	Scoliosis	542	Pneumonia	670
6	Respiratory failure	45 515	Sickle cell disease with crisis	23 261	Pneumonia	479	Extreme immaturity (birth weight, 500-749 g)	668
7	Major depressive disorder	43 156	Partial epilepsy with and without intractable epilepsy	18 635	Major depressive disorder	442	Respiratory distress syndrome in newborn	650
8	Dehydration	39 568	Septicemia	18 588	Acute appendicitis without peritonitis	348	Transposition of great vessels	627
9	Acute appendicitis with peritonitis	37 993	Complications of surgical procedures or medical care	18 416	Cellulitis	327	Respiratory failure of newborn	614
10	Acute appendicitis without peritonitis	37 236	Neutropenia	17 786	Septicemia	316	Tetralogy of fallot	603

Abbreviation: CCC, complex chronic condition.

## Discussion

In this cohort study using a newly developed *ICD-10-CM*–based pediatric grouper and administrative and billing data from 45 tertiary care US children’s hospitals, including more than 2 million inpatient hospital encounters, we provide an updated prioritization of topics for comparative effectiveness research in hospital pediatrics. Much has changed since the initial prioritization study,^6^ including the transition to *ICD-10-CM*, new evidence and treatment protocols, population size and demographics, and costs associated with inpatient stays.^27^ These updated results on prevalence, cost, and variation in cost could be used by funders and the research community as one input to inform comparative effectiveness research prioritization. For example, this data combined with patient, family, and clinician priorities can be used to establish a research agenda in hospital pediatrics.^28,29^ Furthermore, for conditions for which high-quality evidence exists, these data on prevalence and cost can also be used by clinicians and health care administrators to prioritize quality improvement initiatives.

An important finding in our study is the inclusion of 2 mental health conditions among the 50 most costly and prevalent conditions from inpatient encounters, compared with no mental health conditions reported previously.^6^ Major depressive disorder was the 19th most costly and 10th most prevalent condition, while suicide and intentional self-inflicted injury was the 57th most costly and 20th most prevalent. These findings are consistent with other reports on the substantial increase in mental health disorder hospitalizations and costs in children.^30,31,32^ Furthermore, both conditions had high variation in standardized cost, with major depressive disorder having the highest ICC for cost and 33 cost outlier hospitals. The high rank in prevalence and cost of the mental health conditions may also reflect the shortage of inpatient psychiatric facilities. Children who require inpatient mental health treatment are often admitted to the medical unit until a psychiatric inpatient bed becomes available, referred to as *mental health boarding*.^33^ Mental health boarding may result in delays obtaining access to psychiatric inpatient services and lead to long inpatient stays with high encounter costs.^34^ Another contributing factor may be the shortage of child psychiatrists in both outpatient facilities and hospitals in several US regions,^35,36^ with declining ratios of child psychiatrists to children over time.^35^ Poor access to outpatient psychiatric care may result in higher mental health–related hospitalizations. These high costs and variations signal the need for increased research on effective diagnostics and therapeutics for children hospitalized with mental health conditions, increased infrastructure for providing mental health services, greater care standardization and care quality monitoring, and increased availability of inpatient psychiatric services for children.

While a direct comparison between this study and the study by Keren et al^6^ is difficult owing to differences in patient type used to identify priorities and coding (*ICD-9-CM* vs *ICD-10-CM*), there were notable changes in our updated prioritization ranking. Conditions that were ranked higher in cumulative cost in our study included septicemia and respiratory failure in newborns, while conditions that were ranked lower included necrotizing enterocolitis, cellulitis, and cystic fibrosis. We also observed an increase in the interhospital cost variation among some conditions in our study including asthma, respiratory distress syndrome in newborns, dehydration, and acute appendicitis without peritonitis.

We identified the top 25 most prevalent and 25 most costly conditions in children with a CCC vs children without. Children with a CCC accounted for 39.3% of the inpatient encounters and were responsible for substantial costs: the 25 most costly conditions costed $15.8 billion in children with a CCC vs $8.3 billion in children without CCC. Similar findings of high hospital costs in children with medical complexity have been reported previously.^26,37,38^ In some of the most costly conditions found in both groups (eg, respiratory failure, pneumonia), the cost per encounter in children with CCC was 2- to 3-fold higher than in children without CCC. Comparative effectiveness research is needed to inform how to best manage conditions in children with medical complexity, as they are often excluded from clinical trials for common conditions, such as pneumonia and bronchiolitis.^39,40^ Researchers can include children with medical complexity in future studies by including additional safety measures and subgroup analyses. Further, complex care programs that bridge inpatient and outpatient care can reduce hospitalizations, hospital days, and hospital costs in medically complex children.^41,42,43^

### Limitations

This study has some limitations. First is the possible misclassification of conditions owing to coding errors with administrative data or varying coding practices across hospitals, which may be one source of variation in costs. Second, standardized costs using Cost Master Index^6^ do not reflect the true costs of providing care but rather interprets the volume of resources consumed during the encounter. Standardized costs may also make costs at hospitals with lower internal costs incorrectly appear higher than their actual cost, and vice versa.^21^ Nevertheless, standardized cost, which uses the same unit prices across hospitals, is a valuable approach for understanding variation in resource use. Future research could use time-driven activity-based costing, which estimates the cost of resources consumed as a patient moves along a care process to more accurately estimate cost.^44,45^ Third, it is possible that unmeasured factors (eg, unmeasured comorbidities) account for some of the interhospital variation in costs. Our analyses serve to identify conditions that require further research to understand the sources of variation (eg, clinical management) and drivers of interhospital differences in resource use (eg, lack of evidence or lack of care standardization despite high-quality evidence). Future condition-specific research could drill down using secondary diagnosis codes to understand variation in cost across hospitals. Fourth, the 30% quintile-based approach used to identify outlier hospitals may seem arbitrary; however, there is currently no criterion standard or standard threshold. The approach used in this study was based on a previous study by Keren et al.^6^ Fifth, the PHIS database does not include data from community hospitals, and it will be important to conduct similar analyses using data from community hospitals. Sixth, there are variations across hospitals in disease severity, operative complexity, and availability of resources for conditions, and this may affect the variation in costs. Seventh, this study also did not include data from during the COVID-19 pandemic, which has been associated with significantly reduced pediatric hospitalization volume.^46^ Eighth, burden of illness (ie, cost, prevalence) was used to identify conditions that should be prioritized for research in hospital pediatrics. There are other important inputs, such as clinician and patient priorities,^47,48,49^ and other research-related criteria (eg, cost and time required to complete the research) that are critical for identifying research priorities.^12^

## Conclusion

In this cohort study, we provide an updated prioritization list of conditions for comparative effectiveness research in hospital pediatrics using information on prevalence, cost, and variation in cost of hospitalizations at 45 US children’s hospitals. Comparative effectiveness research is important for determining which clinical interventions, such as diagnosis and treatment protocols, and health care delivery models are most effective in improving health outcomes in the real-world setting. The results of our study could assist funders and researchers to develop and refine a research agenda in hospital pediatrics and assist clinicians and health care administrators to prioritize quality improvement initiatives.

## Notes

Supplement 1. **eFigure.** Cohort Flow Diagram **eTable 1.** Prevalence and Cost of Top 25 Most Prevalent and 25 Most Costly Hospital Conditions in Children with Complex Chronic Conditions (CCC) at 45 US Children’s Hospitals, 2016 to 2019 **eTable 2.** Prevalence and Cost of Top 25 Most Prevalent and 25 Most Costly Hospital Conditions in Children without Complex Chronic Conditions (CCC) at US Children’s Hospitals, 2016 to 2019

Supplement 2. **Network Collaborators.** Pediatric Research in Inpatient Setting (PRIS) Network

## References

[1] Lassman D, Hartman M, Washington B, Andrews K, Catlin A. US health spending trends by age and gender: selected years 2002-10. Health Aff (Millwood). 2014;33(5):815-822. doi:10.1377/hlthaff.2013.1224 24799579

[2] Bui AL, Dieleman JL, Hamavid H, . Spending on children’s personal health care in the United States, 1996-2013. JAMA Pediatr. 2017;171(2):181-189. doi:10.1001/jamapediatrics.2016.4086 28027344 PMC5546095

[3] Cohen E, Uleryk E, Jasuja M, Parkin PC. An absence of pediatric randomized controlled trials in general medical journals, 1985-2004. J Clin Epidemiol. 2007;60(2):118-123. doi:10.1016/j.jclinepi.2006.03.015 17208117

[4] Groff ML, Offringa M, Emdin A, Mahood Q, Parkin PC, Cohen E. Publication trends of pediatric and adult randomized controlled trials in general medical journals, 2005–2018: a citation analysis. Children (Basel). 2020;7(12):293. doi:10.3390/children7120293 33333770 PMC7765242

[5] Institute of Medicine. Initial National Priorities for Comparative Effectiveness Research. The National Academies Press; 2009. doi:10.17226/12648

[6] Keren R, Luan X, Localio R, ; Pediatric Research in Inpatient Settings (PRIS) Network. Prioritization of comparative effectiveness research topics in hospital pediatrics. Arch Pediatr Adolesc Med. 2012;166(12):1155-1164. doi:10.1001/archpediatrics.2012.1266 23027409

[7] Centers for Disease Control and Prevention. *International Classification of Diseases, Ninth Revision, Clinical Modification* (*ICD-9-CM*). Accessed June 17, 2021. https://www.cdc.gov/nchs/icd/icd9cm.htm

[8] Monestime JP, Mayer RW, Blackwood A. Analyzing the ICD-10-CM transition and post-implementation stages: a public health institution case study. Perspect Health Inf Manag. 2019;16(Spring):1a. 31019430 PMC6462880

[9] Centers for Disease Control and Prevention. *International Classification of Diseases, Tenth Revision, Clinical Modification* (*ICD-10-CM*). Accessed June 17, 2021. https://www.cdc.gov/nchs/icd/icd10cm.htm

[10] Cartwright DJ. ICD-9-CM to ICD-10-CM codes: what? why? how? Adv Wound Care (New Rochelle). 2013;2(10):588-592. doi:10.1089/wound.2013.0478 24761333 PMC3865615

[11] Institute of Medicine Committee on Priorities for Assessment and Reassessment of Health Care Technologies; Donaldson MS, Sox HC Jr, eds. Setting Priorities for Health Technologies Assessment: A Model Process. National Academies Press; 1992. doi:10.17226/2011 25144089

[12] Dubois RW, Graff JS. Setting priorities for comparative effectiveness research: from assessing public health benefits to being open with the public. Health Aff (Millwood). 2011;30(12):2235-2242. doi:10.1377/hlthaff.2011.0136 22147850

[13] Office of the Assistant Secretary for Planning and Evaluation. U.S. Federal Poverty Guidelines used to determine financial eligibility for certain federal programs. Accessed October 21, 2020. https://aspe.hhs.gov/poverty-guidelines

[14] Washington State Department of Health. Guidelines for using rural-urban classification systems for community health assessment. Revised October 2016. Accessed October 21, 2020. https://www.doh.wa.gov/Portals/1/Documents/1500/RUCAGuide.pdf

[15] Peltz A, Wu CL, White ML, . Characteristics of rural children admitted to pediatric hospitals. Pediatrics. 2016;137(5):e20153156. doi:10.1542/peds.2015-3156 27244794 PMC4845869

[16] WWAMI Rural Health Research Center. RUCA data: using RUCA data. Accessed October 28, 2020. https://depts.washington.edu/uwruca/ruca-uses.php

[17] Feudtner C, Feinstein JA, Zhong W, Hall M, Dai D. Pediatric complex chronic conditions classification system version 2: updated for ICD-10 and complex medical technology dependence and transplantation. BMC Pediatr. 2014;14(1):199. doi:10.1186/1471-2431-14-199 25102958 PMC4134331

[18] Peltz A, Hall M, Rubin DM, . Hospital utilization among children with the highest annual inpatient cost. Pediatrics. 2016;137(2):e20151829. doi:10.1542/peds.2015-1829 26783324 PMC9923538

[19] Gill PJ, Anwar MR, Thavam T, Hall M, Rodean J, Mahant S. Pediatric Clinical Classification System for use in inpatient settings. JAMA Pediatr. 2021;175(5):525-527. doi:10.1001/jamapediatrics.2020.6007 33646300 PMC7922237

[20] Agency for Healthcare Research and Quality, Healthcare Cost and Utilization Project. Clinical classifications software refined (CCSR). Accessed November 27, 2019. https://www.hcup-us.ahrq.gov/toolssoftware/ccsr/ccs_refined.jsp

[21] Mahant S, Richardson T, Keren R, Srivastava R, Meier J; Pediatric Research in Inpatient Setting (PRIS) Network. Variation in tonsillectomy cost and revisit rates: analysis of administrative and billing data from US children’s hospitals. BMJ Qual Saf. Published online June 20, 2020. doi:10.1136/bmjqs-2019-010730 32606211

[22] US Bureau of Labor Statistics. Consumer Price Index for all urban consumers: hospital and related services in U.S. city average. Accessed January 13, 2021. https://fred.stlouisfed.org/series/CUUR0000SEMD

[23] Tieder JS, McLeod L, Keren R, ; Pediatric Research in Inpatient Settings Network. Variation in resource use and readmission for diabetic ketoacidosis in children’s hospitals. Pediatrics. 2013;132(2):229-236. doi:10.1542/peds.2013-0359 23878044

[24] Cameron DB, Graham DA, Milliren CE, . Quantifying the burden of interhospital cost variation in pediatric surgery: implications for the prioritization of comparative effectiveness research. JAMA Pediatr. 2017;171(2):e163926. doi:10.1001/jamapediatrics.2016.3926 27942727

[25] Jonas JA, Shah SS, Zaniletti I, . Regional variation in standardized costs of care at children’s hospitals. J Hosp Med. 2017;12(10):818-825. doi:10.12788/jhm.2829 28991947

[26] Cohen E, Berry JG, Camacho X, Anderson G, Wodchis W, Guttmann A. Patterns and costs of health care use of children with medical complexity. Pediatrics. 2012;130(6):e1463-e1470. doi:10.1542/peds.2012-0175 23184117 PMC4528341

[27] Agency for Healthcare Research and Quality, Healthcare Cost and Utilization Project. HCUP fast stats—trends in inpatient stays. Accessed January 14, 2021. https://www.hcup-us.ahrq.gov/faststats/NationalTrendsServlet?measure1=03&characteristic1=01&time1=10&measure2=&characteristic2=01&time2=10&expansionInfoState=hide&dataTablesState=hide&definitionsState=hide&exportState=hide

[28] James Lind Alliance Priority Setting Partnerships. Paediatric hospital care (Canada). Accessed May 6, 2021. https://www.jla.nihr.ac.uk/priority-setting-partnerships/paediatric-hospital-care-canada/

[29] Harrison JD, Archuleta M, Avitia E, . Developing a patient- and family-centered research agenda for hospital medicine: the Improving Hospital Outcomes through Patient Engagement (i-HOPE) study. J Hosp Med. 2020;15(6):331-337. doi:10.12788/jhm.3386 32490806 PMC7289507

[30] Bardach NS, Coker TR, Zima BT, . Common and costly hospitalizations for pediatric mental health disorders. Pediatrics. 2014;133(4):602-609. doi:10.1542/peds.2013-3165 24639270 PMC3966505

[31] Doupnik SK, Lawlor J, Zima BT, . Mental health conditions and medical and surgical hospital utilization. Pediatrics. 2016;138(6):e20162416. doi:10.1542/peds.2016-2416 27940716 PMC5127076

[32] Zima BT, Rodean J, Hall M, Bardach NS, Coker TR, Berry JG. Psychiatric disorders and trends in resource use in pediatric hospitals. Pediatrics. 2016;138(5):20160909. doi:10.1542/peds.2016-0909 27940773 PMC5079078

[33] McEnany FB, Ojugbele O, Doherty JR, McLaren JL, Leyenaar JK. Pediatric mental health boarding. Pediatrics. 2020;146(4):e20201174. doi:10.1542/peds.2020-1174 32963020

[34] Claudius I, Donofrio JJ, Lam CN, Santillanes G. Impact of boarding pediatric psychiatric patients on a medical ward. Hosp Pediatr. 2014;4(3):125-132. doi:10.1542/hpeds.2013-0079 24785553

[35] McBain RK, Kofner A, Stein BD, Cantor JH, Vogt WB, Yu H. Growth and distribution of child psychiatrists in the United States: 2007-2016. Pediatrics. 2019;144(6):e20191576. doi:10.1542/peds.2019-1576 31685696 PMC6889947

[36] Findling RL, Stepanova E. The workforce shortage of child and adolescent psychiatrists: is it time for a different approach? J Am Acad Child Adolesc Psychiatry. 2018;57(5):300-301. doi:10.1016/j.jaac.2018.02.008 29706155

[37] Neff JM, Sharp VL, Muldoon J, Graham J, Myers K. Profile of medical charges for children by health status group and severity level in a Washington State Health Plan. Health Serv Res. 2004;39(1):73-89. doi:10.1111/j.1475-6773.2004.00216.x 14965078 PMC1360995

[38] Berry JG, Hall M, Neff J, . Children with medical complexity and Medicaid: spending and cost savings. Health Aff (Millwood). 2014;33(12):2199-2206. doi:10.1377/hlthaff.2014.0828 25489039 PMC5164920

[39] Mahant S, Wahi G, Bayliss A, ; Canadian Paediatric Inpatient Research Network (PIRN). Intermittent vs continuous pulse oximetry in hospitalized infants with stabilized bronchiolitis: a randomized clinical trial. JAMA Pediatr. 2021;175(5):466-474. doi:10.1001/jamapediatrics.2020.6141 33646286 PMC7922227

[40] Livingston MH, Mahant S, Connolly B, . Effectiveness of intrapleural tissue plasminogen activator and dornase alfa vs tissue plasminogen activator alone in children with pleural empyema: a randomized clinical trial. JAMA Pediatr. 2020;174(4):332-340. doi:10.1001/jamapediatrics.2019.5863 32011642 PMC7042898

[41] Mosquera RA, Avritscher EBC, Pedroza C, . Hospital consultation from outpatient clinicians for medically complex children: a randomized clinical trial. JAMA Pediatr. 2021;175(1):e205026-e205026. doi:10.1001/jamapediatrics.2020.5026 33252671 PMC7783544

[42] Feudtner C, Hogan AK. Identifying and improving the active ingredients in pediatric complex care. JAMA Pediatr. 2021;175(1):e205042-e205042. doi:10.1001/jamapediatrics.2020.5042 33252681

[43] Casey PH, Lyle RE, Bird TM, . Effect of hospital-based comprehensive care clinic on health costs for Medicaid-insured medically complex children. Arch Pediatr Adolesc Med. 2011;165(5):392-398. doi:10.1001/archpediatrics.2011.5 21300650

[44] Keel G, Savage C, Rafiq M, Mazzocato P. Time-driven activity-based costing in health care: a systematic review of the literature. Health Policy. 2017;121(7):755-763. doi:10.1016/j.healthpol.2017.04.013 28535996

[45] Kaplan RS, Anderson SR. Time-driven activity-based costing. Harv Bus Rev. 2004;82(11):131-138, 150. doi:10.5117/mab.82.12839 15559451

[46] Gill PJ, Mahant S, Hall M, Berry JG. Reasons for admissions to US children’s hospitals during the COVID-19 pandemic. JAMA. 2021;325(16):1676-1679. doi:10.1001/jama.2021.4382 33904877 PMC8080216

[47] Hoffman JM, Keeling NJ, Forrest CB, . Priorities for pediatric patient safety research. Pediatrics. 2019;143(2):e20180496. doi:10.1542/peds.2018-0496 30674609 PMC6361358

[48] Morris C, Simkiss D, Busk M, . Setting research priorities to improve the health of children and young people with neurodisability: a British Academy of Childhood Disability-James Lind Alliance Research Priority Setting Partnership. BMJ Open. 2015;5(1):e006233. doi:10.1136/bmjopen-2014-006233 25631309 PMC4316435

[49] Vella-Baldacchino M, Perry DC, Roposch A, . Research priorities in children requiring elective surgery for conditions affecting the lower limbs: a James Lind Alliance Priority Setting Partnership. BMJ Open. 2019;9(12):e033233. doi:10.1136/bmjopen-2019-033233 31892663 PMC6955494

