# Supplementary material for: Identifying Conditions With High Prevalence, Cost, and Variation in Cost in US Children’s Hospitals
Source: JAMA Netw Open. 2021 Jul 26;4(7):e2117816. doi: 10.1001/jamanetworkopen.2021.17816 (PMC8314139; doi:10.1001/jamanetworkopen.2021.17816)
Supplement: Supplement 1. — eFigure. Cohort Flow Diagram eTable 1. Prevalence and Cost of Top 25 Most Prevalent and 25 Most Costly Hospital Conditions in Children with Complex Chronic Conditions (CCC) at 45 US Children’s Hospitals, 2016 to 2019 eTable 2. Prevalence and Cost of Top 25 Most Prevalent and 25 Most Costly Hospital Conditions in Children without Complex Chronic Conditions (CCC) at US Children’s Hospitals, 2016 to 2019 [file jamanetwopen-e2117816-s001.pdf]

Supplemental Online Content

Gill PJ, Anwar MR, Thavam T, et al; Pediatric Research in Inpatient Setting (PRIS) Network. Identifying conditions with high prevalence, cost, and variation in cost in US children's hospitals. *JAMA Netw Open*. 2021;4(7):e2117816. doi:10.1001/jamanetworkopen.2021.17816

eFigure. Cohort Flow Diagram

eTable 1. Prevalence and Cost of Top 25 Most Prevalent, and 25 Most Costly Hospital Conditions in Children with Complex Chronic Conditions (CCC) at 45 US Children's Hospitals, 2016 to 2019

eTable 2. Prevalence and Cost of Top 25 Most Prevalent, and 25 Most Costly Hospital Conditions in Children without Complex Chronic Conditions (CCC) at US Children's Hospitals, 2016 to 2019

This supplemental material has been provided by the authors to give readers additional information about their work.

© 2021 Gill PJ et al. *JAMA Network Open.*

eFigure. Cohort Flow Diagram

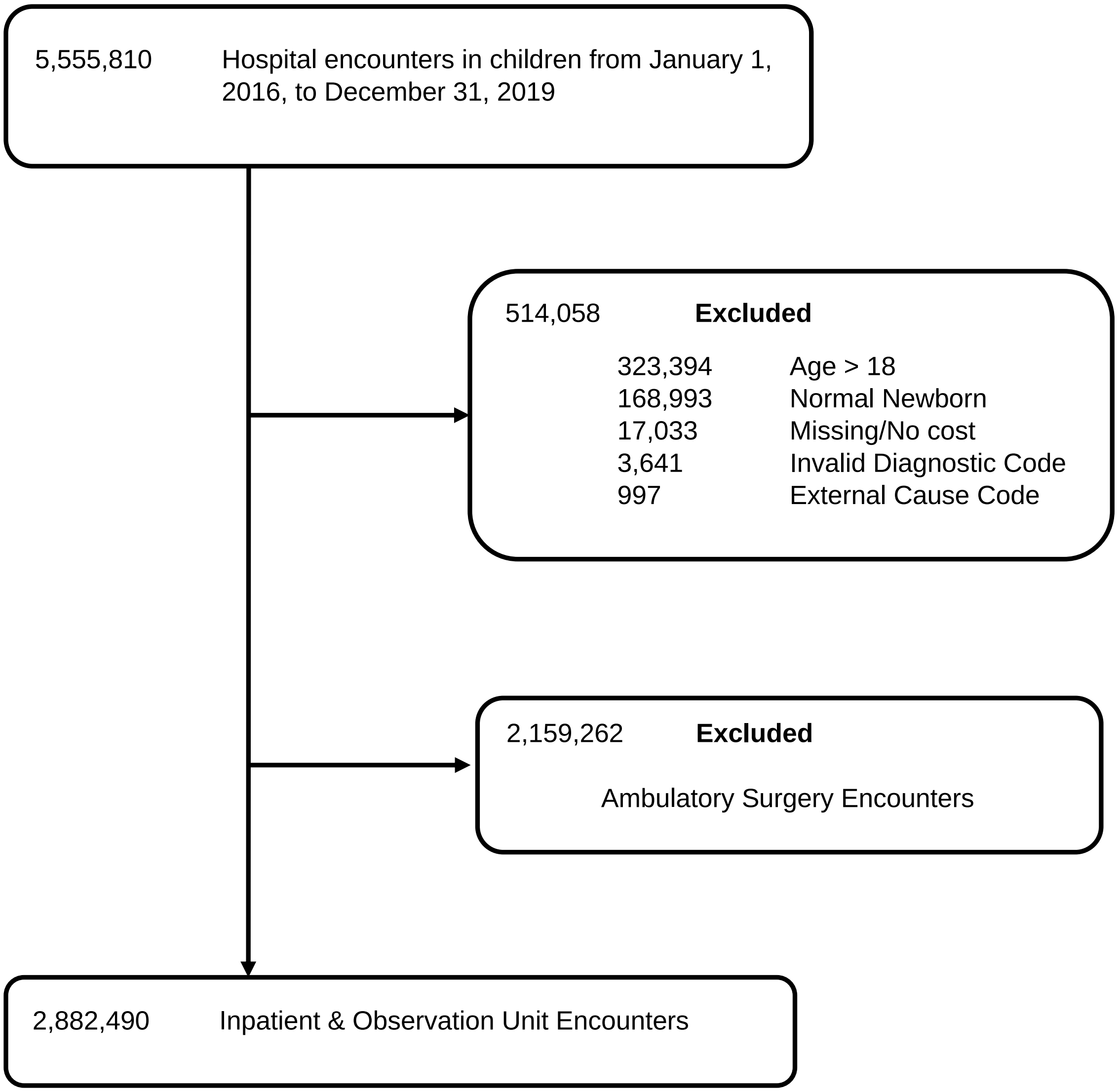

© 2021 Gill PJ et al. *JAMA Network Open.*

eTable 1. Prevalence and Cost of Top 25 Most Prevalent, and 25 Most Costly Hospital Conditions in Children with Complex Chronic Conditions (CCC) at 45 US Children's Hospitals, 2016 to 2019

	Rank Based On			Standardized Cost (USD)	
**Condition**	**Cost**	**Prevalence**	**N**	**Per Encounter**	**Total, Millions**
Chemotherapy	1	1	70727	24557	1737
Respiratory failure	2	2	33981	49058	1667
Septicemia	3	8	18588	65452	1217
Hypoplastic left heart syndrome	4	49	5373	155749	837
Pneumonia	5	4	28416	23580	670
Extreme immaturity (500-749 grams)	6	138	1745	382910	668
Respiratory distress syndrome in newborn	7	74	3776	172258	650
Transposition of great vessels	8	53	5026	124821	627
Respiratory failure of newborn	9	64	4077	150521	614
Tetralogy of Fallot	10	36	6558	91978	603
Complications of surgical procedures or medical Care	11	9	18416	31487	580
Congestive heart failure (non-hypertensive)	12	91	3081	183635	566
Extreme immaturity (750-999 grams)	13	141.5	1699	321494	546
Scoliosis	14	28	7449	68147	508
Acute lymphoid leukemia without remission	15	42	6197	71577	444
Coarctation of aorta or interrupted aortic arch	16	51	5227	82204	430
Bronchopulmonary dysplasia	17	164	1496	278224	416
Anomalies of diaphragm, congenital	18	128	1931	212366	410
Endocardial cushion defects, other	19	71	3841	100320	385
Cystic fibrosis	20	17	9530	40408	385
Seizures with and without intractable epilepsy	21	3	31313	12100	379
Neutropenia	22	10	17786	20771	369
Necrotizing enterocolitis	23	181	1309	280750	368
Sepsis of newborn	24	107	2689	136079	366
Gastroschisis & exomphalos	25	124	2114	172017	364
Bronchiolitis	26	5	23693	15078	357
Complication of implant or graft device	27	13	13881	24821	345
Sickle cell disease with crisis	30	6	23261	13298	309
Partial epilepsy with and without intractable epilepsy	31	7	18635	16569	309
Other nutritional; endocrine; and metabolic disorders	47	20	9147	22929	210
Other nervous system disorders	54	25	8206	22398	184
Crohn's disease	59	19	9194	18572	171
Dehydration	65	12	15305	10538	161
Diabetic ketoacidosis	67	11	16140	9336	151

© 2021 Gill PJ et al. *JAMA Network Open.*

Urinary tract infections	68	14	12669	11816	150
Cardiac dysrhythmias	69	22	8668	16952	147
Gastroenteritis, Infectious	73	15	10771	12157	131
Viral infection	89	16	9974	11223	112
Acute upper respiratory infection	102	18	9406	9504	89
Constipation	111	24	8323	9644	80
Asthma	115	21	9032	8761	79
Fever of unknown origin	120	23	8409	9049	76

Abbreviations: N, total number of encounters; USD, United States dollar.

© 2021 Gill PJ et al. *JAMA Network Open.*

eTable 2. Prevalence and Cost of Top 25 Most Prevalent, and 25 Most Costly Hospital Conditions in Children without Complex Chronic Conditions (CCC) at US Children's Hospitals, 2016 to 2019

	**Rank Based On**			**Standardized Cost (USD)**	
**Condition**	**Cost**	**Prevalence**	**N**	**Per Encounter**	**Total, Millions**
Bronchiolitis	1	1	119686	7329	877
Respiratory failure	2	6	45515	15529	707
Asthma	3	2	108642	6087	661
Acute appendicitis with peritonitis	4	9	37993	15809	601
Scoliosis	5	43	9380	57828	542
Pneumonia	6	3	55468	8630	479
Major depressive disorder	7	7	43156	10246	442
Acute appendicitis without peritonitis	8	10	37236	9352	348
Cellulitis	9	4	48133	6799	327
Septicemia	10	30	12730	24806	316
Respiratory distress syndrome in newborn	11	74	4688	64338	302
Fracture of lower limb	12	12	23266	12045	280
Dehydration	13	8	39568	6518	258
Hypertrophy of tonsils and adenoids	14	5	46813	4803	225
Preterm infants (2000-2499 grams)	15	67	5443	41005	223
Seizures with And without intractable epilepsy	16	11	26507	7398	196
Intracranial injury	17	39	9915	17499	174
Complications of surgical procedures or medical care	18	22	17178	10040	172
Suicide and intentional self-inflicted injury	19	13	23170	7434	172
Preterm newborn	20	83	4188	41057	172
Infective arthritis and osteomyelitis	21	40	9832	16476	162
Preterm infants (1750-1999 grams)	22	119	2731	57645	157
Urinary tract infections	23	18	21249	7319	156
Gastroenteritis, infectious	24	17	21760	7104	155
Preterm infants (1500-1749 grams)	25	158	1859	81764	152
Specified conditions originating in perinatal period	26	24	16539	9169	152
Supracondylar fracture of humerus	27	16	21996	6660	146
Fracture of upper limb	30	23	16557	8452	140
Other Convulsions	35	14	23087	5230	121
Constipation	37	21	17394	6681	116
Viral infection	38	20	18033	6419	116
Abdominal pain	44	25	16370	5907	97
Neonatal hyperbilirubinemia	46	15	22235	4199	93
Croup	57	19	20663	3529	73

Abbreviations: N, total number of encounters; USD, United States dollar.

© 2021 Gill PJ et al. *JAMA Network Open.*
