# Supplementary material for: Identifying Conditions With High Prevalence, Cost, and Variation in Cost in US Children’s Hospitals
Source: JAMA Netw Open. 2021 Jul 26;4(7):e2117816. doi: 10.1001/jamanetworkopen.2021.17816 (PMC8314139; doi:10.1001/jamanetworkopen.2021.17816)
Supplement: Supplement 2. — Network Collaborators. Pediatric Research in Inpatient Setting (PRIS) Network [file jamanetwopen-e2117816-s002.pdf]

Supplemental Online Content: Nonauthor Collaborators

*Indicates required information. Only first name, last name, and suffix will appear in PubMed.

***Group Name(s): Pediatric Research in Inpatient Setting (PRIS) Network**							
***First Name and Middle Initial(s)**	***Last Name**	***Suffix (eg, Jr, III)**	Academic Degrees	Institution	Location (city, state/province, country)	Role or Contribution, eg, chair, principal investigator	Group (if more than 1 Group listed in the byline) and/or Subgroup (eg, Steering Committee)
Peter J	Gill		MD DPhil	Hospital for Sick Children	Toronto	Co-Principal Investigator	
Matt	Hall		PhD	Children's Hospital Association	Lenexa, Kansas	Co-Investigator	
Jonathan	Rodean		MPP	Children's Hospital Association	Lenexa, Kansas	Co-Investigator	
Sunitha	Kaiser		MD MSc	University of California, San Fransisco	San Fransisco, California	Co-Investigator	
Rajendu	Srivastava		MD MPH	Intermountain Health Inc	Salt Lake City, Utah	Co-Investigator	
Ron	Keren		MD MPH	Children's Hospital of Philadelphia	Philadelphia, PA	Co-Investigator	
Sanjay	Mahant		MD MSc	Hospital for Sick Children	Toronto	Principal Investigator	
